# Development of a Hydrogen Gas Sensor Using a Double Saw Resonator System at Room Temperature

**DOI:** 10.3390/s150304749

**Published:** 2015-02-26

**Authors:** Zainab Yunusa, Mohd Nizar Hamidon, Alyani Ismail, Maryam Mohd Isa, Mohd Hanif Yaacob, Saeed Rahmanian, Siti Azlida Ibrahim, Arafat A.A Shabaneh

**Affiliations:** 1 Institute of Advanced Technology, Universiti Putra Malaysia, 43400 Serdang, Selangor, Malaysia; E-Mail: zee2yunusa@gmail.com; 2 Department of Electrical Engineering, Bayero University Kano, P.M.B 3011 Kano, Nigeria; 3 Department of Computer and Communications, Universiti Putra Malaysia, 43400 Serdang, Selangor, Malaysia; E-Mails: alyani@.upm.edu.my (A.I.); hanif@upm.edu.my (M.H.Y.); azlida@mmu.edu.my (S.I.); tamimi_127@hotmail.com (A.A.S.); 4 Department of Electrical and Electronics Engineering, Faculty of Engineering, Universiti Putra Malaysia 43400 Serdang, Selangor, Malaysia; E-Mail: maryam@upm.edu.my; 5 Department of Mechanical and Manufacturing Engineering, Faculty of Engineering Universiti Putra Malaysia, 43400 Serdang, Selangor, Malaysia; E-Mail:saeedfed@gmail.com; 6 Faculty of Engineering, Multimedia University, 63100 Cyberjaya, Selangor, Malaysia

**Keywords:** double SAW resonator, hydrogen gas sensor, polyaniline and carbon nanotubes

## Abstract

A double SAW resonator system was developed as a novel method for gas sensing applications. The proposed system was investigated for hydrogen sensing. Commercial Surface Acoustic Wave (SAW) resonators with resonance frequencies of 433.92 MHz and 433.42 MHz were employed in the double SAW resonator system configuration. The advantages of using this configuration include its ability for remote measurements, and insensitivity to vibrations and other external disturbances. The sensitive layer is composed of functionalized multiwalled carbon nanotubes and polyaniline nanofibers which were deposited on pre-patterned platinum metal electrodes fabricated on a piezoelectric substrate. This was mounted into the DSAWR circuit and connected in parallel. The sensor response was measured as the difference between the resonance frequencies of the SAW resonators, which is a measure of the gas concentration. The sensor showed good response towards hydrogen with a minimum detection limit of 1%.

## 1. Introduction

One of the main sources of air pollution comes from power generation as a result of the fossil fuel consumed by the power plants. In order to reduce fossil fuel consumption, the interest in using hydrogen as a clean energy source or a fuel gas has been increased remarkably because it is renewable, abundant and efficient, with zero emissions. Hydrogen is also used extensively in some industries to make ammonia, methanol and rocket fuel and also as a replacement for natural gas in warming homes and powering hot water heaters [1,2,3]. The first priority in using hydrogen gas as fuel like other gas fuels is safety: hydrogen is flammable and potentially dangerous. The explosive limit of hydrogen is more than 4% [4] so careful handling, storage and transportation are required. The monitoring of the concentration is very important to avoid accidents resulting from hydrogen explosions and therefore, a reliable, sensitive and selective gas sensor is required for this job. Surface acoustic wave (SAW) technology has been one of the technologies used to meet these requirements and at the same time, due to its low power consumption, it can be used wirelessly.

Basically, the SAW technology can be configured into two different configurations, which are SAW delay lines and SAW resonators, that are proved to be promising for the hydrogen gas sensing application [5,6,7,8,9,10,11]. The SAW delay line is employed if the interest is to observe the time response of the sensor where the sensitive layer been deposited on the active delay area between the interdigital transducer (IDT). Meanwhile the SAW resonators are preferred when the sensor response is based on the resonance frequency by depositing the active layer on to IDT of the resonator itself. In this paper, the focus was more on the resonator rather than the delay line because we were concerned with controlling the resonance frequency of the sensor rather than its time response. Gas sensing using a single SAW resonator is commonly employed and has proven reliable for the detection of different gases [12,13,14,15] in which the sensor response is expressed as upshift or downshift of resonance frequency. The double SAW resonator (DSAWR) is another type of sensing configuration which has been used by some researchers for direct and remote sensing. This technique has been demonstrated by [16,17,18] for sensing applications, including strain and temperature sensors, where it has proved to be reliable, but it has never been employed for gas sensing application to the best knowledge of the authors. In this paper a DSAWR system been developed as a novel system for gas sensing other than the conventional method that uses single resonator, as discussed earlier.

Carbon nanotubes (CNT) have been proved to be good sensing materials for gas detection due to their inherent properties like good electrical conductivity and their hollow structure. However, due to their highly metallic behaviour they caused short circuits in the IDTs. In order to deposit the sensing layer successfully, an insulating/guiding layer which needs to be sandwiched between the sensing material and the electrodes has to be created. This could be seen in the work of Penza *et al.* [19], where a guiding layer made up of SiO_2_ was deposited in between the substrate and the carbon nanotubes to fabricate a sensor for vapour detection based on a two-port resonator. In another work by the same author [20], a ZnO/LiTaO_3_ was used as a substrate and ZnO acts as a guiding layer for protecting the IDTs before a single walled carbon nanotubes nanocomposites layer is deposited on the resonator. Similarly, David *et al*. [14] reported that depositing multiwalled carbon nanotubes (MWNTs) directly on the IDTs caused shorts and this was overcome by mixing the MWNTs with cerium oxide which will reduce their metallic behaviour. Therefore, it is of interest to investigate a novel less cumbersome technique for deposition of the sensing material which can prevent the IDTs from short circuiting as well as eliminate the fabrication of a guiding layer for SAW resonators. Here a new technique is proposed to address the aforementioned problems by fabricating and connecting independently the sensitive layer to the DSAWR system that has been developed. The system will be tested with different hydrogen gas concentrations and the differences between the two shifted resonance frequencies will be used to express the sensor response signal.

## 2. Experimental Section

### 2.1. Design and Fabrication of the DSAWR

**Figure 1:**
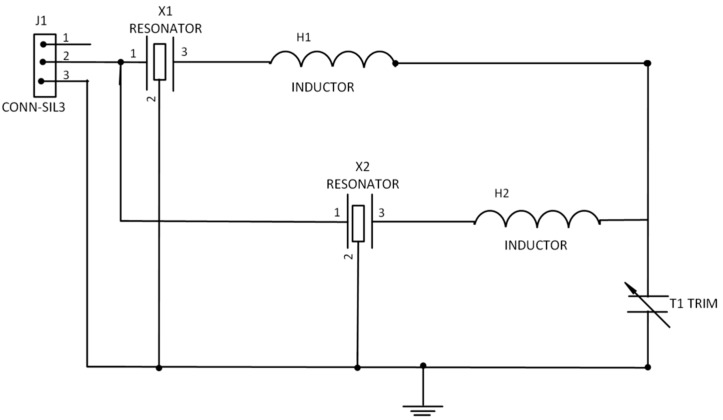
Schematic of DSAWR circuitry.

Commercial SAW one-port resonators with resonance frequencies of 433.42 MHz and 433.92 MHz were used to design the matching circuit. The resonators were first connected to the Anritsu VNA so as to obtain the S-parameters. An L-matching circuit was used to match the resonators to the load due to its simplicity. In order to design the matching circuit, analytical method and simulation was employed. The lumped equivalent circuit was simulated using Sonnet software based on the values obtained from the analytical solution. Figure 1 depicts the schematic diagram for the DSAWR on a printed circuit board (PCB). 

Resonators X1 and X2 were matched with inductors L1 and L2. The variable capacitor acts as a tuning element. The circuit of Figure 1 which was adopted from [18] was designed and fabricated on a PCB. In order to select the appropriate values of the lumped elements of the L-matching network, Equations (1) and (2) were used:

(1) $$B=\pm \frac{1}{Z_{0}}\sqrt{\frac{Z_{0-}R_{L}}{R_{L\;}}}$$

(2) $$X=\pm \sqrt{R_{L}\left(Z_{0}-R_{L}\right)}-X_{L}$$

where *R_L_* is the load resistance and *Z*_0_ is the output impedance whose value is 50 Ω and *X_L_* is the inductive reactance. Once *B* and *X* are calculated the values of L and C in the matching network could be obtained. Measurements of the fabricated circuit were done using an Anritsu Voltage Network Analyzer (Agilent Technologies, CA, USA). Subsequently, the circuit of Figure 1 was also fabricated onto a PCB board and measurements were also carried out.

### 2.2. Active Layer Fabrication

**Figure 2:**
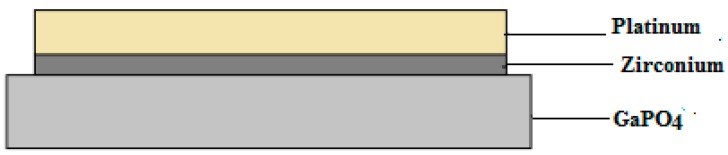
Top view illustration of the substrate.

In order to fabricate the active layer, a piezoelectric substrate was employed so as to initiate the SAW wave propagation. Platinum of 110 nm thickness was chosen as the preferred metal for the electrodes; in between the substrate and the electrodes a thin layer (21 nm) of zirconium was sandwiched so as to promote adhesion between the metal and the substrate. The electrodes were fabricated using electron beam lithography. There were 100 interdigited transducers with a finger width of 1.3 μm. Figure 2 shows an illustration of the top view of the substrate.

### 2.3. Double SAW Resonator Fabrication

**Figure 3:**
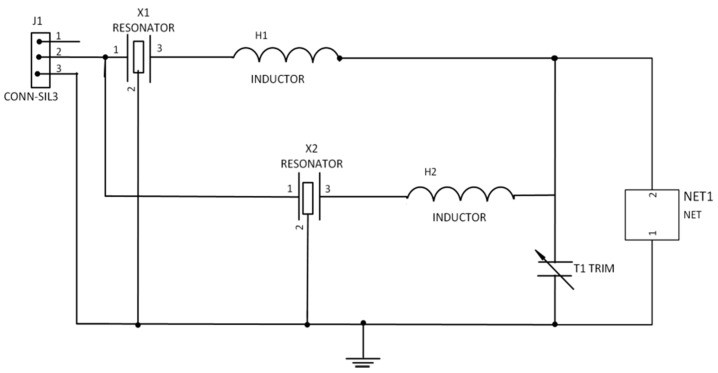
Schematic of DSAWR system with a resistor connected in parallel.

The DSAWR system shown in Figure 3 was fabricated on a printed circuit board. The circuit of Figure 1 was modified and the sensing layer was connected in parallel which is denoted by NET1 as shown in Figure 2. 

**Figure 4:**
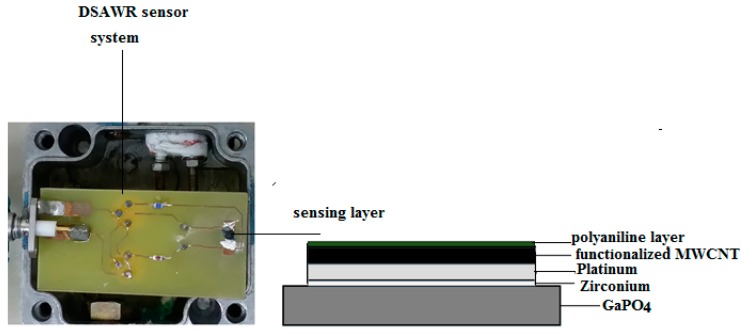
(**a**) Fabricated DSAWR sensor with the sensing layer integrated into the system placed in a test cell; (**b**) Side view of the sensing layer (thickness not to scale).

The reason why the sensing layer was placed in parallel with the capacitor is due to the advantage of isolating it further away from the matching circuit so as to protect it from any disturbances. Tests and measurements were conducted using a FieldFox RF Analyzer N9912A (Agilent technologies, CA, USA) connected to the SMA connector in the DSAWR circuitry. The complete DSAWR system after fabrication is as shown in Figure 4a.

### 2.4. Preparation of Sensitive Layer

#### 2.4.1. Functionalization of Multiwalled Carbon Nanotubes

In order to enhance the sensitivity, functionalization of the MultiWalled Carbon Nanotubes (MWCNT) was done so as to introduce OOH groups. Commercial MWCNT (0.5 g) supplied by the Hangzhou Company (Hangzhou, China) which were produced with a diameter between 20 to 40 nm by catalytic chemical vapor deposition were mixed with concentrated nitric acid and sulphuric acid in the ratio of 3:1. The detailed procedure for the functionalization process can be found in [21].

#### 2.4.2. Preparation of Polymer

Chemical polymerization was employed for the synthesis of emeraldine-based polyaniline. Details of the polymerization process can be found in [22]. After the polymerization process 15 mg of polyaniline powder was mixed with 15 mg of camphorsulphonic acid and dissolved in 8 mg of chloroform so as to produce a green solution of CSA-doped polyaniline with a concentration of 3.75 mg/mL. The solution was then stirred using a magnetic stirrer for 1 h.

#### 2.4.3. Integration of Sensitive Layer unto the Substrate

After functionalization of the commercial MWCNT, 0.2 g was dissolved in 50 mL of acetone and sonicated using an ultrasonic bath for 1 h. The prepared solution was then drop-casted using a micro-pipette unto the piezoelectric substrate with pre-patterned platinum electrodes. The substrate was then dried in the oven at 70 °C for 1 h. After drying, 10 μL of the sonicated CSA-doped polyaniline was sprayed unto the coated substrate with the coated CNT using an airbrush under argon flow. A thin layer of polyaniline was formed on the functionalized MWCNT layer. The DSAWR system as shown in Figure 4a contains all the electrical components which includes the inductors and capacitor for the matching of the two resonators and the sensing layer while, the inset of Figure 4a shows the side view of our sensor as shown in Figure 4b. After deposition of the sensitive layer, Figure 2 is modified and has a layer of functionalized CNT and another layer of polyaniline; these two layers form the sensitive layer for hydrogen detection.

#### 2.4.4. D-Experimental Gas Measurement Set-Up

After preparation of the sensing layer, it was mounted on the PCB and integrated into the DSAWR system. Frequency measurements using a FieldFox RF Analyzer N9912Aportable network analyser were used to monitor the gas sensor response by observing the resonant frequency shifts from the two resonators.

**Figure 5:**
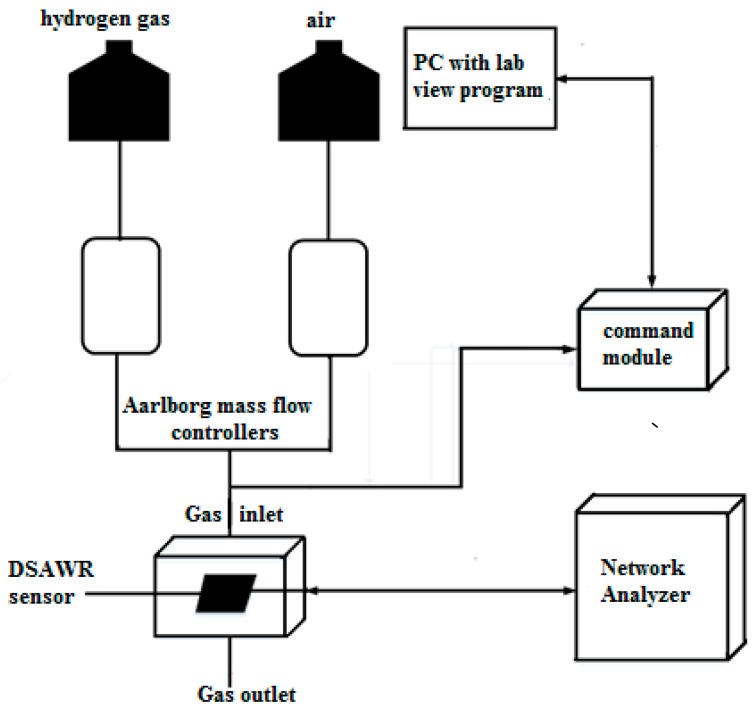
Gas sensing measurement set-up schematic.

The gas sensing measurement set-up which is illustrated in Figure 5 consists of computerized Aalborg mass flow controllers which control synthetic air and hydrogen gas at a flow rate of 0.2 mL/min.

The mass flow controllers are connected to the hydrogen gas and air. A command module was connected to a computer and controlled by an automated Labview program that controls the gas sensing process. Measurements were carried out with a portable Fieldfox network analyser connected to the SMA connector of the DSAWR system. Synthetic air was allowed to flow into the system for 30 min to stabilize the system. All testing were carried out at 25 °C which is the ambient room temperature. Subsequently a process of gas injection and desorption was repeated for different concentrations of hydrogen ranging between 1% and 2%. The same cycle of 10 min hydrogen gas injection and 10 min air desorption was maintained throughout the sensing experiment.

## 3. Results and Discussion

### 3.1. Double SAW Resonator

**Figure 6:**
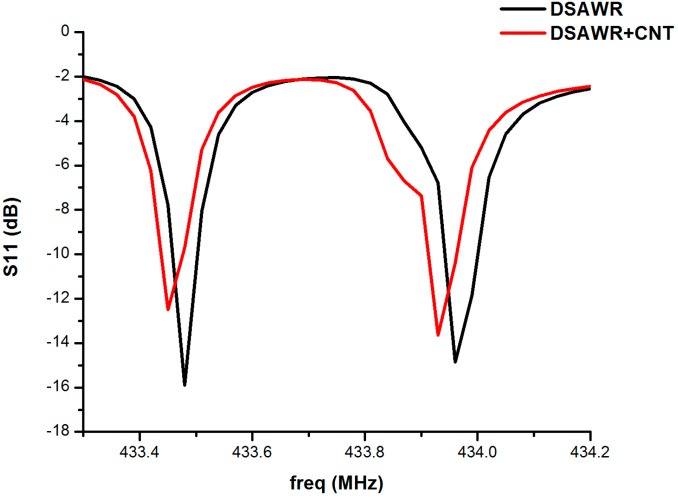
Plot of S11 *versus* frequency.

The responses of the DSAWR when connected to the vector network analyser (VNA) after fabrication of the circuit in Figure 1 and Figure 2 is as shown in Figure 6. A plot of S11 *versus* resonance frequency is shown with two resonant frequencies corresponding to the commercial SAW resonators used in the fabrication. The response is also shown as displayed by the VNA. The blue curve represents the response obtained when the circuit of Figure 1 was connected to the VNA.

It has resonance frequencies at 433.48 and 433.99 MHz, respectively, while the insertion losses are −15.89 and −14.86 dB respectively. However, from the datasheet of the manufacturer the minimum and maximum resonant frequencies for resonator 1 are 433.42 and 433.495 MHz, respectively, while those of resonator 2 are 433.845 and 433.995 MHz, respectively, so the results obtained are still within the tolerance values. The difference in the resonant frequencies could be as a result of parasitic effects and losses due to soldering. When the fabricated circuit of Figure 2 was connected to the VNA the resonant frequencies decreased to 433.45 and 433.96 MHz, respectively, while the insertion losses also decreased to −12.51 and −13.65 dB, respectively. The decrease in resonant frequency is as a result of the mass loading effect due to the integration of the sensing layer into the DSAWR. The resulting mass effect decreases the acoustic velocities of the SAW resonator and this results in a decrease in the resonant frequencies.

### 3.2. Scanning Electron Microscope

**Figure 7:**
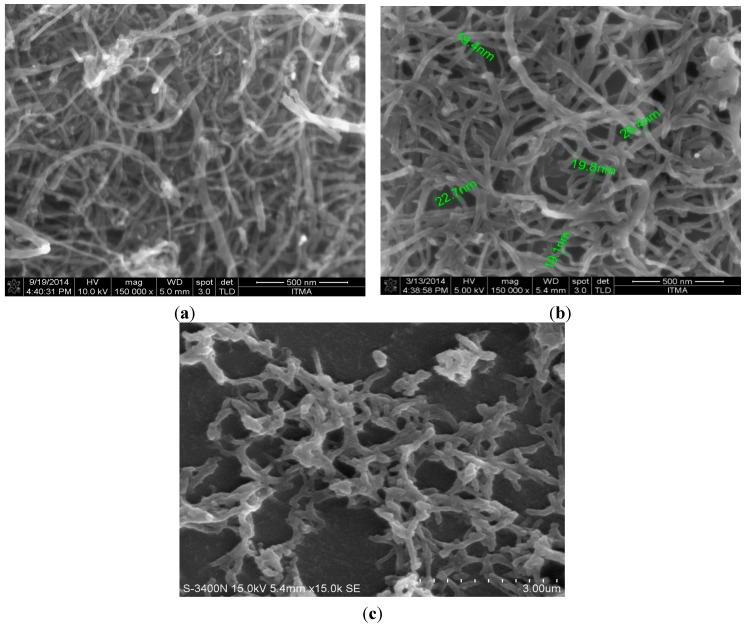
(**a**) SEM micrograph of MWNT; (**b**) SEM micrograph of functionalized CNT; (**c**) SEM of Polyanilinenanofibers.

A Scanning Electron Microscope was used to characterize the synthesized pristine, functionalized CNT and the polyaniline nanofibers. The diameters range from 20 to 40 nm as shown in Figure 7a while Figure 7b shows the SEM of functionalized CNT as seen the morphology and the structure is similar, which means the acid treatment did not affect the surface morphology of the CNT. However, it could be seen that the CNT are debundled, which shows that the acid treatment was successful. The aim of the acid treatment is to debundle the CNT which are densely packed as shown in Figure 7a so as to allow free interaction and attraction between the CNT and the gas molecules.

The hollow structure of the CNT allows for the adsorption of the gas molecules and the resistance of the CNT will change upon interaction. The CSA-doped polyaniline was deposited unto a glass substrate in order to see the morphology. Similarly, Figure 7c shows the morphology structure of the polyaniline. It could be observed that is relatively homogeneous with average diameters of about 300 nm.

### 3.3. Raman Spectroscopy

**Figure 8:**
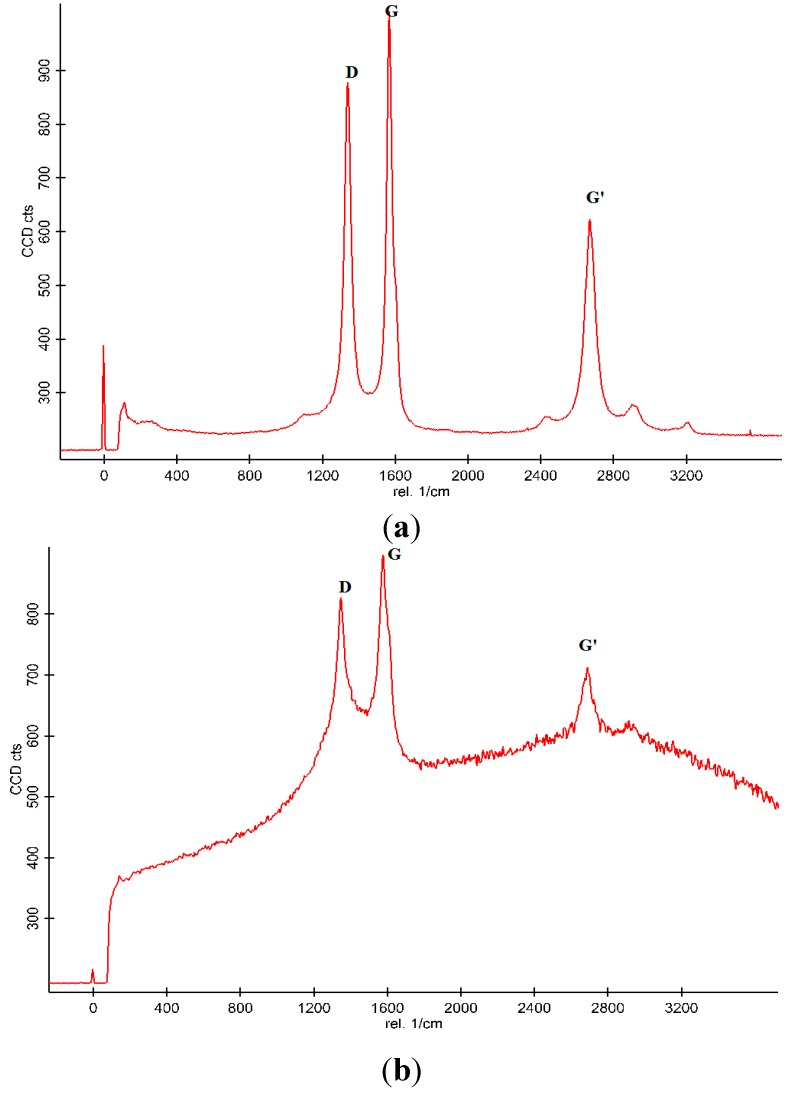
(**a**) Raman spectroscopy of Pristine MWCNT; and (**b**) Functionalized MWCNT.

Raman spectroscopy was employed as a tool for the qualitative analysis of pristine-MWCNT and functionalized CNT as shown in Figure 8a and Figure 8b. As seen from Figure 8a prominent peaks, namely the D-band, G-band and D+G bands are located at 1350, 1600 and 2700 cm^−1^, respectively. The D-band is a measure of the disorder of the carbon structures in the tubes, while the G-band is a measure of the graphitic mode and it is as a result of vibration as a result of the stretching of the sp^2^ molecules. The Id to Ig ratio is a measure of crystallinity and inversely proportional [23]. The Raman spectrum is in agreement with Raman spectroscopy of MWCNTs [24]. Figure 8b shows the Raman spectrum of acid-treated CNTs. After functionalization it could be observed that the intensity of the peaks increases due to the introduction of OOH groups. The Id to Ig ratio for the pristine CNT was found to be 0.85 while that of the functionalized CNT was found to be 0.93. The increase in the ratio of the D-band to the G-band is due to the introduction of the functional group which causes defects in the walls of the CNT. It could be observed that the functionalization does not affect the inherent structure of the pristine CNT as seen from the Raman spectrum, but the intensity of the G-band increases in the functionalized CNT from. This increase could be as a result of increased conductivity due to the presence of functional group as reported by [25]. The introduction of functional groups enhances the Raman signal due to the loosely bounded tubes and defective walls. Since gas sensing is based on adsorption/desorption of the hollow structure of the nanotubes, it was anticipated that the functionalization will lead to improved sensitivity due to the random movement of particles and its interaction with the target gas as the result of the defective walls.

### 3.4. Fourier Transform InfraRed (FTIR)

**Figure 9:**
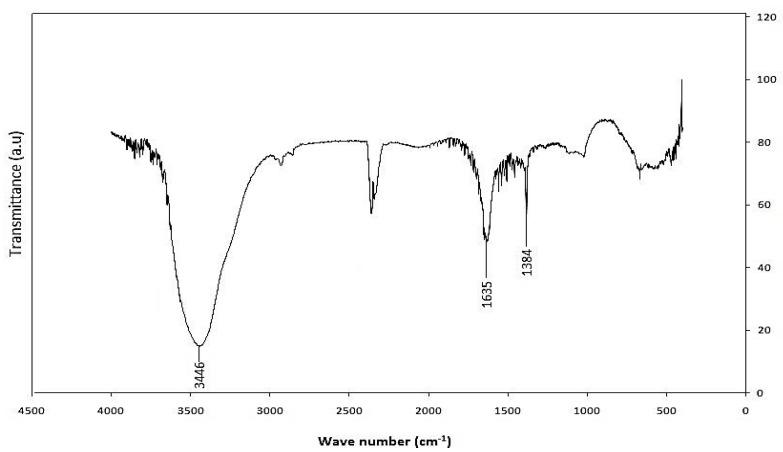
FTIR spectrum of functionalized-CNTs.

FTIR was employed as a tool for the determination of the presence of functional groups. The FTIR spectrum for functionalized MWCNT is shown in Figure 9. A pronounced broad peak at ~3440 cm^−1^ which corresponds to OH stretching is seen. An absorption peak at ~2359 cm^−1^ was also observed and it is associated with C-H stretch modes. The characteristic carbonyl peak is also observed at about 1636 cm^−1^ and can be assigned to the carbonyl group from quinine or ring structures [26,27]. The bands in the region of 1380 cm^−1^ can be ascribed either to carboxyl—carbonate structures or to aromatic C=C bonds and various substitution modes of the aromatic ring [28].

### 3.5. Hydrogen Gas Sensing Results

**Figure 10:**
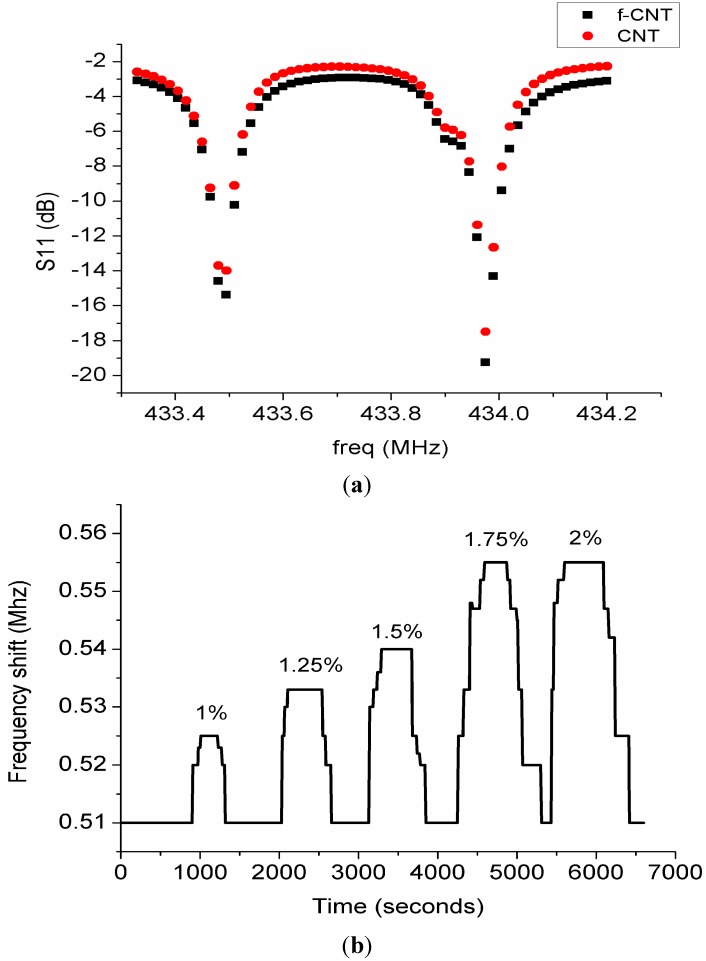
(**a**) Response of CNT and functionalized CNT to hydrogen gas; (**b**) Plot of frequency shift *versus* time for different hydrogen gas concentrations.

The gas sensor response was observed and the results were plotted for various concentrations of hydrogen. Figure 10 shows a plot of hydrogen concentration *versus* time. The response of the gas was measured as the difference in resonance frequencies of the two resonators denoted by Δf. It has been established that the gas sensor response is a measure of frequency shift of the resonator as a result of the interaction of gas molecules with the sensing layer. The mechanism of the sensor response using single SAW resonator is that frequency shifts as a result of upshift or downshift of the resonance frequency are observed.

In this paper we used a double SAW resonator so as to investigate its potential for wireless sensing applications. Our sensor response is therefore based on the difference between the two resonance frequencies as a result of gas adsorption/desorption. The principle of the mechanism in SAW gas sensing is that any changes in mass density, elastic modulus or electrical conductivity of the sensing layer directly affect the acoustic wave propagation. This in turn affects the velocity of propagation which invariably affects the resonance frequency. This is expressed by the relationship:

(3) $$\frac{\Delta f}{f}=\frac{\Delta v}{v}$$

where Δf is defined as frequency shift. In the DSAWR system:

(4) $$\Delta f=f_{2}-f_{1}$$

where $f_{2}$ is the resonance frequency of SAW resonator 2 and $f_{1}$ is the resonance frequency of SAW resonator 1. Frequency shifts were only observed for hydrogen concentrations between 1% and 2% as shown in Figure 10. For this system the frequency shift has a limited bandwidth for the upper and lower frequency difference based on the tolerance values as highlighted in the data sheet of the SAW resonators. It was observed concentrations below 1% do not produce significant changes in ∆f and this could be explained based on the behaviour of our system which has a limited bandwidth of resonance frequency. Preliminary results using the substrate with platinum electrodes only showed no sensor response. Figure 10 shows the response of the sensor after it was connected to the analyser, air was purged in for 30 min to stabilize the system. Hydrogen gas was then introduced into the chamber with the help of the mass flow controllers for 10 min and subsequently air was purged in for another 10 min. This process was repeated for different variations of hydrogen gas concentrations between 1% and 2%. It was observed that there was no shift in Δf throughout the process of gas adsorption and desorption as shown in Figure 10a. This could be explained by the lack of affinity of pristine carbon nanotubes towards hydrogen at room temperature. As reported by [29] sensing of hydrogen was only observed at high temperature between 150 °C and 300 °C. Recently another group investigated the use of MWNT sheets for hydrogen detection at room temperature, and higher concentrations of hydrogen from 6% to 18% were detected at room temperature but the recovery required annealing the sensor at a temperature of 100 °C [25]. In order to achieve sensing at room temperature, functionalization of CNT with OH radical, noble metal nanoparticles or CNT nanocomposites which includes metal oxides or polymers are required [30,31,32]. We tried to functionalize the pristine CNT with an OH radical group by using acids but when the sensor was tested towards hydrogen there was no sensor response as shown in Figure 10a. The authors then decided to use nanocomposites of CNT with polyaniline nanofibers as it was reported earlier by Arsat *et al*. that polyaniline nanofibers are a good sensing material for hydrogen detection. It was observed that neither of them gave a sensor output when exposed to hydrogen because there was no change in ∆f during both gas adsorption and desorption. This is because pristine MWNTs lack much affinity to hydrogen at room temperature. Functionalized MWNTs also did not give us any response at room temperature and it is speculated that the reason due to incomplete neutralization of the acids. However, in order to perform the sensing at room temperature since SAW is a passive device, the authors proposed to use polyaniline nanofibers as shown by [33] as a promising sensitive layer for hydrogen detection.

The response of our sensor when exposed to hydrogen with MWCNT/polyaniline is shown in Figure 10b. The system was tested for different variations of hydrogen gas concentration between 1% and 2%. It could be observed that when the sensor was exposed to different hydrogen concentrations there is a decrease in conductivity of the functionalized MWCNT/polyaniline layer which causes an increase in acoustic velocity and then an increase in resonant frequency [33]. When the sensor was exposed to 1% hydrogen concentration the response time was about 300 s and the frequency shift increases gradually until it reaches a maximum saturated value of 0.525 while the recovery time was observed to be 120 s. The exposure time for both hydrogen and synthetic air were maintained at a fixed time of 600 s. The percentage of hydrogen gas was increased gradually for the same period for gas adsorption and desorption. It was observed that the frequency shift increases as the gas concentration increases. The high percentage concentration of 2% has the fastest response time of 40 s. However recovery times increase as the hydrogen gas concentration increases due to the increased mass adsorped by the sensitive layer. The lowest hydrogen percentage concentration of 1% has the fastest recovery time of 120 s while for full concentration of hydrogen gas (2%) the recovery time was approximately 420 s. It could also be observed however that the frequency shift of 1.75% and 2% hydrogen concentrations is the same, but the response times and recovery times are different, which means that the sensor has attained saturation. The response time of 1.75% was only 60 s while that of 2% hydrogen concentration was only 40 s. It could be observed that our plots shown in Figure 10b have some step-like behaviour. This is due to the fact that the response observed is not a continuous waveform but rather more digital-like and the sensor response was measured as the difference between the frequency shifts of the two resonators.

The increase in the frequency shifts are attributed to the high surface to volume ratio of the polyaniline nanofibers. The response of our sensor to CSA-doped polyaniline could be supported by the work of [34] in which the interactions of hydrogen gas with doped and dedoped polyaniline nanofibres were investigated. It was reported that the doped polyaniline gave a better response to hydrogen gas than the dedoped polyaniline. Dedoped polyaniline presents low conductivity therefore doping is required so as to increase the conductivity. The higher the conductivity the faster it is for the molecules to attract each other and interact freely. In this work, the dedoped polyaniline powder was doped with CSA. This was chosen for increased conductivity as well as better dispersion of the polyaniline powder in the chloroform solution. Similarly, It was also reported by [33] that frequency shifts were observed for different concentrations of hydrogen gas between 0.5% to 1% at room temperature.

The response of the functionalized CNT/polyaniline sensitive layer could also be explained based on the interactions of the hydrogen ions with the functionalized-CNT. As shown by [25] hydrogen sensing using functionalized CNT was achieved at room temperature. The sensitivity of the sensor could therefore be explained by the effect of the presence of functional groups which causes dissociation of hydrogen gas molecules into hydrogen atoms at the edges of the functional groups. Thus, defective edges serve as a catalytic site for the adsorption of hydrogen molecules.

**Figure 11:**
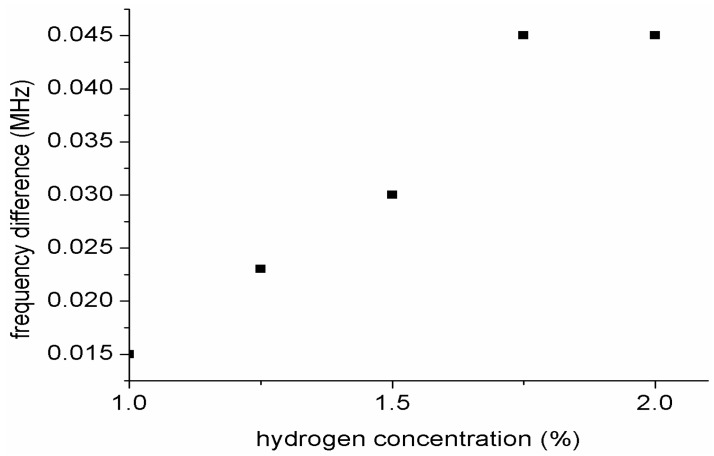
Plot of frequency shift *versus* hydrogen gas concentration.

Figure 11 shows a plot of frequency shift *versus* hydrogen gas concentration. From the graph the sensitivity of the sensor towards hydrogen concentration was calculated to be 3.2 Hz/ppm. It could be noted that the sensitivity of our sensor is not high. This is because our sensor response is based on frequency shift between the two resonators as such there is a limited bandwidth for the resonance frequencies. The frequency shift was obtained based on the difference between the difference between the frequency shift of each gas concentration and the frequency gas of the reference gas which is synthetic air.

## 4. Conclusions

A DSAWR was successfully fabricated and applied for hydrogen gas detection. Commercial resonators were employed for the DSAWR system. The IDTs were fabricated with platinum metal electrodes and a piezoelectric substrate. The DSAWR was tested towards hydrogen gas of concentrations between 1% and 2% at room temperature. Different sensing materials were used so as to investigate the most suitable configuration of hydrogen sensing. Preliminary results using pristine CNT and functionalized CNT did not yield any sensor response. Response to hydrogen was only observed with functionalized CNT and polyaniline nanofibers at room temperature which is one of the advantages of conductive polymers. The limit of detection for our sensor was 1% therefore future work needs to focus on how to reduce the detection limit by making ∆f more sensitive. Other sensitive layers that have very strong affinity towards hydrogen gas need to be tested with the proposed system. The sensor response which was measured as a difference between the two resonant frequencies showed a good response towards hydrogen gas. Response time was observed to be 40–420 s, while recovery time was in the range of 120–420 s, depending on the gas concentration. The higher the gas concentration, the longer the recovery time of the sensor. It was observed that as the gas concentration increases the frequency shift also increases which is due to the mass changes as a result of conductivity changes of the sensitive layer. The sensitivity of the sensor was measured to be 3.2 Hz/ppm. However one of the limitations of the sensor is instability which is due to the polyaniline nanofibers and has been one of the limitations of polymer-based gas sensors. Therefore, future work needs to focus on regeneration of the polyaniline so as to obtain good stability.
